# Syncytium calcium signaling and macrophage function in the heart

**DOI:** 10.1186/s13578-018-0222-6

**Published:** 2018-03-27

**Authors:** Xin Zhou, Zhongguang Li, Zefan Wang, Eda Chen, Juan Wang, Frederic Chen, Odell Jones, Tao Tan, Shawn Chen, Hiroshi Takeshima, Joseph Bryant, Jianjie Ma, Xuehong Xu

**Affiliations:** 1 Laboratory of Cell Biology, Genetics and Developmental Biology, Shaanxi Normal University College of Life Sciences, Xi’an, 710062 People’s Republic of China; 2 Ohio State University College of Medicine, Columbus, OH 43210 USA; 3 Virginia Commonwealth University College of Medicine, Richmond, VA 23284 USA; 4 Chen Wellness Clinics, Wichita, KS 67219 USA; 5 University of Pennsylvania ULAR, Philadelphia, PA 19144 USA; 6 Graduate School of Pharmaceutical Sciences, Kyoto University, Kyoto, 606-8501 Japan; 7 Institute of Human Virology, University of Maryland School of Medicine, Baltimore, MD 21287 USA

**Keywords:** Macrophage, Electrical connection, Calcium dependency, Colony-stimulating factors (CSF), Mononuclear phagocyte system (MPS)

## Abstract

Macrophages are traditionally viewed as a key component of the immunity defense system. Recent studies have identified resident macrophages in multiple organs including the heart, in which the cells perform their crucial role on tissue repair after myocardial infarction (MI). The cardiac-specific macrophages interdigitate with cardiomyocytes particularly at the atrioventricular node region. The integrative communication between macrophage and cardiomyocytes can modulate the contractile function of the heart. Coordinated control of intracellular calcium signaling and intercellular electrical conduction via the syncytium network underlie the synchronized beating of the heart. In this review article, we introduce the concept the syncytium calcium signaling in the cardiomyocytes can modulate gene expression in the resident macrophages and their integration with the cardiomyocytes. The cardiac macrophages originate from bone marrow stem cells, migrate to local via vessel, and settle down as a naturalization process in heart. As the macrophages perform on regulating electrical conduction, and accomplish post MI non-scared completed regeneration or partial regeneration with fibrotic scar at different stage of postnatal development, we understand that multiple functions of cardiac macrophage should carry on with diverse linages. The naturalization process in heart of macrophages to the cardiomyocytes serves important roles to control of electrical signaling and calcium-dependent contractile function of the heart.

## Introduction

As a major component in the first line of immunity defense, macrophages are distributed in almost every tissues, including cardiac macrophages in the heart, cerebellum microglia cells in the brain, hepatic Kupffer cells in the liver, alveolar macrophages in the lungs, and Langerhans cells in the epidermis. Although macrophage functions in the immune system have been investigated extensively [1–3], the tissue-specific functions of macrophages in the heart are largely unknown. As the ATM/mTOR signaling, Rac1-GTPase, and PI3 K/AKT pathways play critical roles in controlling migration of cell [1–4], the multiple cell surface antigens such as CCR2/CD192, CD64/FcγR1, CX3CR1 and Mac3 were linked to origination of monocyte-macrophage differentiation and polarization in post myocardial infarction (MI) [5–9], but the molecular basis of macrophages migrating into specific tissues under physiological or pathological conditions, and fundamental knowledge of cell–cell recognition are much more obscure.

The cardiac macrophages developed from bone marrow stem cells (plus spleen stem cells as well in mouse), migrated through cardiac vessel from circulated blood, and settled down with polarization in heart could comprehend as a naturalization process in heart. The macrophages play crucial role on regulating electrical conduction by associating with AV node [10], and they are also critical for post MI repair and recovering of cardiac function after MI. This macrophage associated repair would be accomplished with no-scard completed regeneration in neonatal heart or with a partial regeneration with fibrotic scar after P7 [5, 6]. The cardiac macrophages could perform diverse functions on promoting stem cell-cardiomyocyte regeneration and angiogenesis with different cell linages. Here we propose that control of intracellular calcium signaling contributes to the naturalization process of macrophages in the heart and to modulating the contractile function of the cardiomyocytes in the context of a syncytium network.

## Macrophages facilitate cardiac electrical conduction and promote cardiac regeneration

A recent study by Hulsmans et al. showed that resident macrophages were enriched in human and mouse atrioventricular (AV) node and can regulate the electrophysiological activity of cardiomyocytes through the gap-junction protein, connexin 43 (Cx43), at the “linking” portion of the conducting cardiomyocyte and the macrophage [10, 11]. This pilot study reveals the critical role of tissue-specific macrophages that has never before been recognized in cardiac function, and raises many interesting research subjects about the physiological and pathological bases of human cardiovascular diseases.

**Fig. 1:**
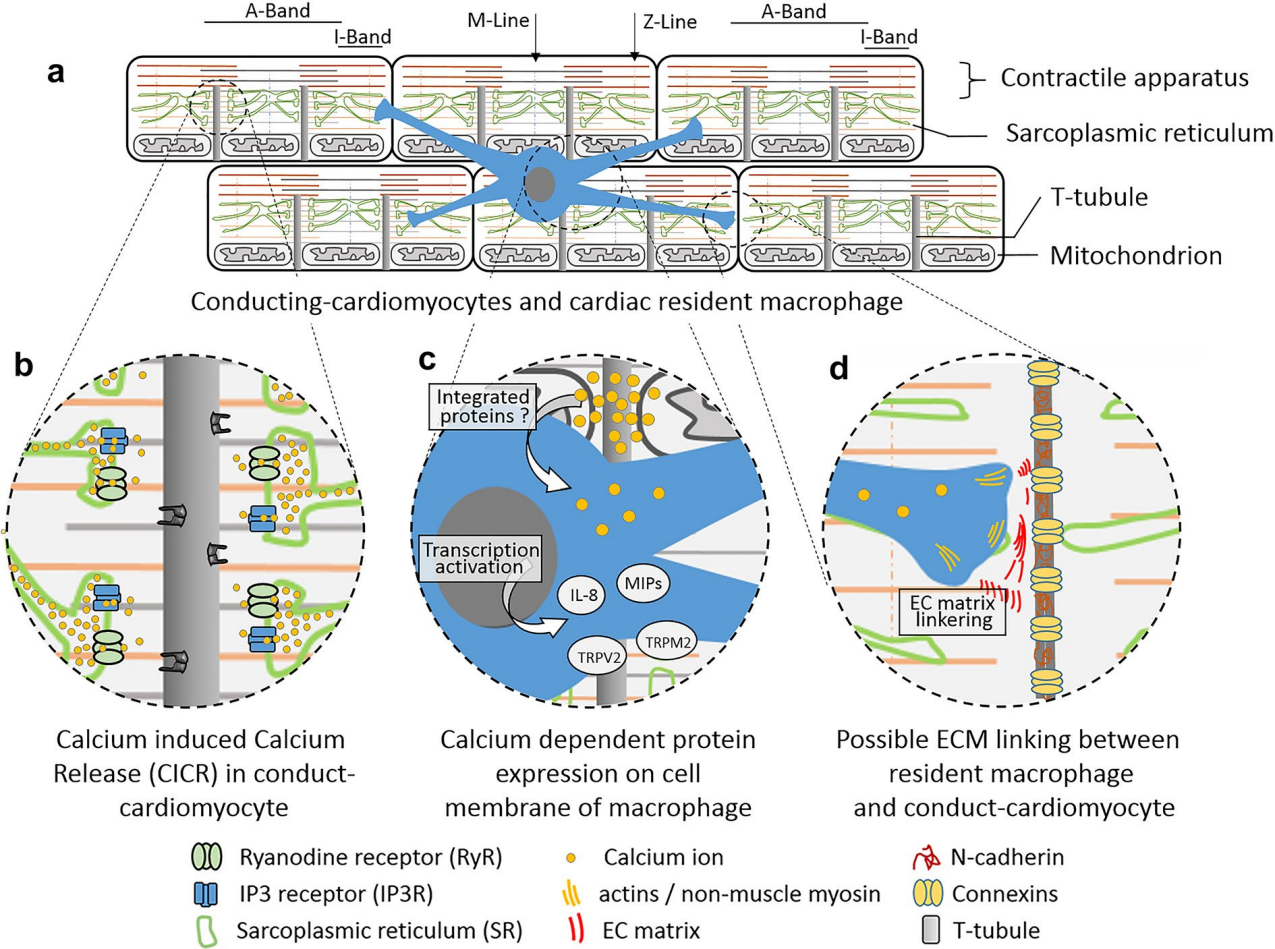
Sketch of the association between resident macrophage and cardiomyocytes in the heart. Cardiomyocytes are aligned longitudinally in the heart (**a**) with actin-myosin apparatus and carry contraction controlled by CICR (**b**). Resident macrophages are naturalized in cardiac tissue (**c**). Through connexin 43 and other integrated and extracellular matrix proteins, resident macrophages facilitate conducting cardiomyocytes and modify the action potential of cardiomyocytes (**d**)

Using GFP labelled cardiac macrophages, cardiomyocytes located in the lower nodal or AV bundle were frequently interspersed with macrophages that have an elongated, spindle-shaped appearance [12, 13]. These macrophages longitudinally distribute along the AV-His bundle, with their cytoplasmic portion extending and reaching cardiomyocytes across long distances [10] (Fig. 1).

In heart tissue, Cx43 is located on the intercalated discs responsible for electrical conduction through neighboring cardiomyocytes, and is essential for the synchronized contraction of the heart. The Cx43-mediated cell–cell linkage provides the connection between resident macrophages and cardiomyocytes [10] and forms the structural framework to couple these two types of cells along with extracellular matrix. Such connection complex could guarantee synchronization of cardiomyocyte contraction and its control by the resident macrophages at the AV His-bundle in the heart.

Although more detailed information on the mechanisms of how resident macrophages facilitate the conduction system in the heart remained to be explored, the physiological function of the macrophages on maintaining AV conduction were supported by several animal model studies where AV blocks were introduced through loss-of-function investigations. With deletion of Cx43 in the macrophages, the *Cx3cr1 Cx43*^−*/*−^ mice developed severe 1st degree and 2nd degree AV block. With CSF1 deletion in the macrophages, the *CSF1*^*op*^ mice could develop non-matured macrophages with a lack of normal function and exhibited 1st degree and 2nd degree AV block. Deletion of macrophage Cd11b [12, 14] in the *Cd11b*^*DTR*^ mice produced 1st degree, 2nd degree, and even life threatening 3rd degree AV block [10]. These three experiments proved the function of the cardiac resident macrophages on maintaining normal conduction in the heart.

The recent study by Hulsmans et al. [10] revealed that the external macrophage can bond to the conducting cardiomyocyte in AV node to regulate the electrophysiological activity of the heart via the gap-junction protein, connexin 43 (Cx43). However, before this discovery, the most researches mainly focused on the repair function of macrophages recruited from blood flow circulating [6, 15, 16, 17]. After MI, circulated monocyte-macrophages were recruited by the infarct zone, and then the naturalizing cells play their critical function on cleaning apoptotic death clashes and promoting cardiac stem cell to regenerate cardiomyocyte. Among those studies, the neonatal repair in 7 day post MI revealed an interesting phenomenon that on P7 neonatal heart, the post MI repair carried by macrophages generated no-scar healing. After P7, the repair could perform a partial regeneration and generate fibrotic scar in the MI zone [6]. Severe difficulty on the completed regeneration post MI was detected within the cardiac macrophage knockout animals. These data suggest the critical roles of the resident cardiac macrophage on cardiomyocytes and angiogenesis [6]. We would like to emphasize that more investigation on this no-scar regeneration in this timing frame could provide us more opportunities to unveil detailed molecular mechanism of the naturalization from circulated cell to resident cardiac macrophage through migration and polarization within the heart early development.

## Surface antigens reflect functional complexity of cardiac macrophages

Rationally, cell surface antigens on specified macrophages would be critical for the cell migration, polarization summarized as naturalization in heart although the related molecular mechanisms are still under investigation. These cardiac macrophage antigens plus intracellular markers of the cell are utilized biomarkers for us to discover function of macrophages in cardiac tissues. The cell surface antigens distribute on monocyte-macrophage include CCR2/CD192, CD64/FcγR1, CX3CR1 and Mac3 [5–9]. Some macrophage specific antigens are also distributed in macrophage cytoplasmic location within endosomal/lysosomal compartment, or secreted to extracellular microenvironment along with their cell surface distribution. The representative antigens in this category are CD68/macrosialin, CD163 and Galectin 3/Mac2 [5, 18, 19].

In adult mammals, cardiac macrophages origin from their bone marrow as well as spleen in mouse. While how macrophages differentiating from monocyte during embryonic development is still a mystery (discussed below), many information are discovered from myocardial infarction mouse model. For tracking the macrophage differentiation and settlement in heart after MI, many gating strategies employed with combined different antigens. The combination Ly6ChighCCR2highCX3CR1lowCD62 L+ used to examines classical monocytes [20], and MHCIIlowCCR2+ and Lineage−CD11 b+F4/80lowLy6C+ for cardiac monocyts in mouse model [21, 22]. The CD45+CD11 b+F4/80+CD206− and CD4+CD11 b+F4/80+CD206+ used to detect mouse classic M1 and M2 macrophage [23], and CD45+CD11 b+F4/80+Ly6Clow for Resident cardiac macrophages [22]. The CD45+F4/80+MHC-IIlowCCR2− and CD45+F4/80+MHC-IIhighCCR2− is routine representative for cardiac resident macrophages [6, 22]. Many others were developed for investigating the mechanism of diverse function of macrophage function in heart.

## Syncytium calcium signaling underlies synchronized contractile activity of the heart

Synchronized contractile function of the heart is essential to life. Exactly how resident macrophages in the heart evolved as a fail-safe way to guarantee robust cardiac output under physiological and pathological conditions remains an important area of research.

Calcium (Ca) ions are important second messengers modulating many cellular functions. In the heart, entry of extracellular Ca via Ca channels located on the plasma membrane triggers opening of the ryanodine receptor (RyR) located in the sarcoplasmic reticulum (SR) through Ca-induced Ca release (CICR) [24–27]. The elementary units of Ca release from SR in cardiomyocytes are discreet, localized events known as Ca sparks. Ca sparks are quantal Ca release events that originate from paracrystalline arrays of RyR channels on the SR surface [13, 14, 24]. The discovery of Ca sparks has revolutionized understanding of the physiology and pathophysiology of Ca signaling in the heart.

Synchronized elevation of intracellular Ca triggers contraction of the actin-myosin apparatus by diastolic depolarization, and the crosstalk of electrical conduction between neighboring cardiomyocytes via the interconnection of their intercalated discs through the connexin complex. Longitudinal flow of Ca signaling via the syncytium network characterizes the heart as an efficient circulation pump.

## Macrophage functions in calcium-dependent manner

Although we normally consider that macrophages function as cells in the front line of the immune system, these macrophages also play critical roles in many other aspects, including cardiac electrical activity, wound repair, embryonic development, and many more [1–3]. All these roles can be categorized into three biological processes: migration, endocytosis and phagocytosis. Cytoskeletal regulated migration drives cell movements in tissues and through endothelial cells to their final destinations, where they will carry out functions involved with Capg, Mpp1, Myo1f, Myo5a and Wip1 [4, 27–29]. Endocytosis accomplished by macrophages is a receptor-mediated uptake process for liquids [30]. The internalized materials will interact with diverse receptors such as Alcam, CD9, CD84, Mamdac2, Itgfg3 and Lgals, and are then degraded rapidly after lysosomal fusion. Phagocytosis as a first defense against pathogen attack is defined as the uptake for solid particles about a few micrometer in diameter. Phagocytosis involves recognition of endocytic receptors, vesicle trafficking and protein degradation, carbohydrate/lipid/DNA digestion and many other processes [4, 31–33]. It is obvious that cell surface antigens are important for all three processes, whether for the cells to execute their tasks, or to distinguish which protocol to initiate.

Recent research demonstrated that Ca may contribute to modulation of gene expression in the macrophage. Using monocyte-derived macrophages (MDMs) from patients with chronic obstructive pulmonary disease (COPD), Provost et al. showed that extracellular Ca could enhance phagocytosis and cytokine secretion associated with IL-8, TNF-α, and macrophage inflammatory protein (MIP) subunits MIP-1a and MIP-1b [34]. Additionally, the bacterial challenge of MDMs increased cell surface expression of bacterial recognition receptors, CD16 and MARCO, which led to increased recognition by the macrophage to more potential pathogens, initiating more phagocytosis. This study provides the base for the therapeutic use of Ca to increase macrophage phagocytosis and decrease chronic bacterial infection [34]. It appears that the expression patterns of cell membrane integrated proteins are critical factors that determine how the cells behave (Table 1). Thus, delineating the communication between extracellular Ca homeostasis with intracellular Ca signaling represents an important area of investigation for the tissue-specific function of macrophages.

**Table 1:** Calcium related gene expression and macrophage functions

#	Gene name	Functions of enconded gene	PubMed ID	Chromosome location	Transcript (bp)	CDS (bp)	References
1	MIP-1α	Activating inflammatory response	NM_002983.2	Chr 17:36088256-36090160	813	279	Provost et al. [34]
2	MIP-1β	Activating inflammatory response	NM_002984.3	Chr 17:36103827-36105621	667	279	Provost et al. [34]
3	Toll-like receptor 4 (TLR4)	Activation of TRPC6-dependent calcium signaling mediates endotoxin induced lung vascular permeability and inflammation	NM_021297.3	Chr 9:117704175-117717491	5494	1920	Tauseef et al. [35]
4	STIM1	Mediate extracellular Ca2+ entry	NM_001277961.1	Chr 11:3855703-4093210	4380	2376	Steinckwich et al. [36]
5	Orai1	Mediate extracellular Ca2+ entry	NM_032790.3	Chr 12:121626550-121642040	1496	906	Steinckwich et al. [36]
6	S100A8	As the site of interplay between extracellular Ca2+ entry and intraphagosomal ROS production	NM_001319197.1	Chr 1:1533590032-153422583	546	351	Steinckwich et al. [36]
7	S100A9	As the site of interplay between extracellular Ca2+ entry and intraphagosomal ROS production	NM_002965.3	Chr 1:153357854-153361027	586	345	Steinckwich et al. [36]
8	Transient receptor potential vanilloid 2 (TRPV2)	Participation early phagocytosis and innate immunity	NM_016113.4	Chr 17:16415542-16437003	2829	2295	Link et al. [29]
9	lL-10	Activating immunoreaction	NM_000572.2	Chr 1:206767603-206772494	1629	537	Kelly et al. [37]
	C-type lectin receptor (CLR): Dectin-1	Activation of phagocytosis and cytokine production	NM_197948.2	Chr 12:10116777-10130269	2503	570	Xu et al. [38]
10	Phospholipase Cγ2	Promote Dectin-1-mediated Ca2+ flux and cytokine production	NM_002661.4	Chr 16:81779258-81962693	8707	3798	Xu et al. [39]
11	Mammalian transient receptor potential protein 2 TRPM2	Aggravates inflammation	NM_001320350.1	Chr 21:44350112-44443081	6026	4662	Yamamoto et al. [40]
12	NFATl	Involved in the regulation of cytokine gene expression in T lymphocytes	NM_001291168.1	Chr 2:168476410-168601657	6644	2724	Savignac et al. [41]
13	NFAT2	Involved in the regulation of cytokine gene expression in T lymphocytes	NM_001278669.1	Chr 18:79395772-79529323	5031	2832	Savignac et al. [41]
14	NFAT4	Involved in the regulation of cytokine gene expression in T lymphocytes	NM_004555.3	Chr 16:68085366-68229259	6453	3207	Savignac et al. [41]
15	MEF2D	Involved in the regulation of cytokine gene expression in T lymphocytes	NM_005920.3	Chr 1:156463721-156500842	5996	4186	Savignac et al. [41]
16	DREAM	Involved in the regulation of cytokine gene expression in T lymphocytes	NM_013434.4	Chr 2:95297324-95386077	2928	771	Savignac et al. [41]
17	IL-8	Activating immunoreaction and proinflammatory	NM_001310420.1	Chr Un: 2233607-2236704	1163	312	Tran et al. [42]; Provost et al. [34]
18	TLR (toll-like receptor)-5	Activating immunoreaction	NM_016928.3	Chr 1:223108401-223143282	4277	2577	Tran et al. [42]
19	β2 integrins	Mediate phagocytosis	NM_001303238.1	Chr 21:44885949-44928873	2928	2103	Tran et al. [42]
20	P2Y2	Elicit Ca2+ oscillations activating immunoreaction	NM_176072.2	Chr 11:73200416-73246743	8736	1134	Hanley et al. [43]
21	P2X4	ATP induced a transient depolarization activating immunoreaction	NM_001256796.1	Chr 12:121209861-121234106	2091	1215	Hanley et al. [43]
22	ll-6	Increased transcription of IL-6 activating immunoreaction	NM_000600.4	Chr 7:22725889-22732002	1197	639	Hanley et al. [43]
23	PYK2	Phosphorylation-proinflammatory	NM_001183767.3	Chr XV: 984942-986462	1521	1521	Cuschieri et al. [44]
24	p38	Translocation-proinflammatory	NM_001078490.1	Chr 11:4821238-4824735	3582	1095	Cuschieri et al. [44]
25	NF-kappaB	Translocation-proinflammatory	NM_001319226.1	Chr 4:102501329-102617302	3900	2907	Cuschieri et al. [44]
26	AP-1	Nuclear translocation-proinflammatory	NM_001334400.1	Chr 1:25982294-25986349	1557	705	Cuschieri et al. [44]
27	TNF-alpha	Upregulated-proinflammatory	NM_000594.3	Chr 6: 31575567-31578336	1686	702	Cuschieri et al. [44]; Provost et al. [34]
28	ERK 1/2	Phosphorylation-proinflammatory	*				Cuschieri et al. [44]
29	29 Fc receptor-lgG	Promote receptor-mediated phagocytosis	**				Hishikawa et al. [45]
30	Protein kinase C (PKC)	Promote nonspecific phagocytosis	***				Hishikawa et al. [45]

* ERK 1/2 are not included as mutiple symbols (references: Cuschieri et al. [44])

** Fe receptor-lgG are not included as mutiple symbols (references: Hishikawa et al. [45])

*** Protein kinase C (PKC) are not included as mutiple symbols (references: Hishikawa et al. [45])

Extracellular Ca influxes through plasma membrane Ca channels take the responsibility for the cytoplasmic phagosomal oxidative reaction and inflammatory cytokine reaction [29, 40, 42]. When specific Ca channel inhibitors were applied, cytokine secretion by Ca-mediated endocytosis were inhibited [34]. The immune effectiveness can be improved with elevation of extracellular Ca concentrations in the range of physiologic levels of Ca signaling [46, 47]. In vitro studies with macrophage-like cell lines U937 and MH-S [48] demonstrated that macrophage recognition to elevated Ca involves a sensor zone on the carbohydrate chains of CD43 [49].

Although the Ca-dependent manner of macrophage function was discovered in monocyte-derived macrophages or macrophage-like U937 and MH-S cells, it is possible that the resident macrophages would behavior according to Ca levels in the micro-environment of their niche in the heart tissue.

## Development and differentiation of macrophages require colony-stimulating factor

Macrophages are developed and differentiated from the mononuclear phagocyte system (MPS) [3, 50]. While myeloid progenitor/granulocytes develop to monoblasts, promonocytes and then monocytes migrate into specific tissues, colony-stimulating factors (CSF) can direct differentiation of MPS. These CSFs include macrophage CSF (CSF-1), granulocyte macrophage (GM-CSF) and fms-like tyrosine kinase 3 ligand (Flt3-ligand) [51–53]. The development and differentiation of tissue-specific resident macrophages have many distinct pathways in both normal development and pathological progress.

The characteristics of macrophages with deletion of CSF-1 in the mouse model pinpoint many critical functions of macrophages in somatic differentiation and the development of the pancreas and nervous system in mammal [53, 54]. Genetic ablation of CSF-1 in mice produced infertility in both males and females due to macrophages failing to adapt to the indigenous tissue and failing to settle down as resident macrophage to build the necessary functional architecture of primary reproduction organs and tissues. Resident macrophages are critical in adult individuals and even more imperative during the differentiation process in mouse embryos. This crucial function of macrophages during animal development also contributes to the configuration of the conduction system in the heart [10].

The mononuclear phagocyte lineage differentiating progress is under the control of macrophage CSF, however, no research has reported the direct involvement of Ca signaling with CSF. An earlier data revealed that the concentration of cytosolic Ca pre-incubated with granulocyte–macrophage CSFs can effectively activate an oxidative burst of granulocytes measured with the production of intracellular superoxide (O2^−^) anions [55]. Release of Ca-containing crystals could change extracellular Ca in the micro-environment and potentially enhance macrophage CSF-mediated osteoclastogenesis [56]. These data demonstrate the possibility that CSF plus Ca could re-pattern cell membrane integrated proteins [34]. It is possible that the micro-environment Ca could affect CSF function during tissue settlement of macrophages in organogenesis along with other type of cells.

## Prospect: Ca dependence could be a mechanism of MPS-to-resident macrophage in heart

In the heart, CICR and syncytium cell–cell communication underlie the synchronized contractions of the cardiomyocytes to drive blood circulation throughout the entire body (Fig. 1a, b). Electrical impulses are carried longitudinally through cardiomyocytes linked by N-cadherin, connexins, and other associated proteins [57, 58] (Fig. 1d). As discussed above, resident macrophages can facilitate this electrical conduction within the AV node [10]. If these is any lineage-tracing data to classify the role of resident cardiac macrophages is the valuable question we have to clear in future investigation, the answer could be mysterious up-to-date. As we discussed, more than 30 surface proteins involve in the functional differentiation from blood monocyte to cardiac monocyte, and from circulated macrophage to resident cardiac macrophage. Meanwhile, the P7 non-scar regeneration and conducting signal promotion by macrophages enlighten that multiple lineage of macrophages could exist for these divers functions.

The concept of resident macrophages facilitating electrical conduction in the heart raises many interesting subjects that should be explored further about the role of macrophages in other cardiac functions such as how pre-mononuclear phagocytes differentiate along with conducting cardiomyocytes, what principle role they play during co-developmental architecture, how these resident macrophages function in adult heart, what maintains their role in continuous contracting tissue as a non-contractile cells, and whether anchoring proteins and extracellular matrix proteins are required to direct and connect resident macrophage to conducting cardiomyocyte.

It should not be a coincidence that there is both a Ca-dependency of macrophages and CICR dependency of cardiomyocytes for contraction. The intracellular Ca in both cells should provide coordination for their integration, and the extracellular Ca should provide a micro-environment for homeostasis. The syncytium Ca signaling would allow for a more efficient macrophage niche within the cardiomyocytes and consequently for the synchronized contraction of the heart.

## Abbreviations

AV: atrioventricular
CSF: colony-stimulating factor
MPS: mononuclear phagocyte system
GM-CSF: granulocyte macrophage
CX43: connexin 43
Ca: calcium
RyR: ryanodine receptor
SR: sarcoplasmic reticulum
CICR: Ca-induced Ca release
COPD: chronic obstructive pulmonary disease
MDM: monocyte-derived macrophage
MIP: macrophage inflammatory protein
MI: myocardial infarction
